# Advanced Kidney Volume Measurement Method Using Ultrasonography with Artificial Intelligence-Based Hybrid Learning in Children

**DOI:** 10.3390/s21206846

**Published:** 2021-10-14

**Authors:** Dong-Wook Kim, Hong-Gi Ahn, Jeeyoung Kim, Choon-Sik Yoon, Ji-Hong Kim, Sejung Yang

**Affiliations:** 1 Department of Biomedical Engineering, Yonsei University, Wonju 26494, Korea; kdw1vv@gmail.com (D.-W.K.); kkwa999@yonsei.ac.kr (H.-G.A.); 77imjee@gmail.com (J.K.); 2 Department of Radiology, Gangnam Severance Hospital, Yonsei University College of Medicine, Seoul 06273, Korea; YOONCS58@yuhs.ac; 3 Department of Pediatrics, Gangnam Severance Hospital, Yonsei University College of Medicine, Seoul 06273, Korea

**Keywords:** kidney volume measurement, ultrasonography, image segmentation, artificial intelligence, hybrid learning

## Abstract

In this study, we aimed to develop a new automated method for kidney volume measurement in children using ultrasonography (US) with image pre-processing and hybrid learning and to formulate an equation to calculate the expected kidney volume. The volumes of 282 kidneys (141 subjects, <19 years old) with normal function and structure were measured using US. The volumes of 58 kidneys in 29 subjects who underwent US and computed tomography (CT) were determined by image segmentation and compared to those calculated by the conventional ellipsoidal method and CT using intraclass correlation coefficients (ICCs). An expected kidney volume equation was developed using multivariate regression analysis. Manual image segmentation was automated using hybrid learning to calculate the kidney volume. The ICCs for volume determined by image segmentation and ellipsoidal method were significantly different, while that for volume calculated by hybrid learning was significantly higher than that for ellipsoidal method. Volume determined by image segmentation was significantly correlated with weight, body surface area, and height. Expected kidney volume was calculated as (2.22 × weight (kg) + 0.252 × height (cm) + 5.138). This method will be valuable in establishing an age-matched normal kidney growth chart through the accumulation and analysis of large-scale data.

## 1. Introduction

Kidney size is well correlated with renal function, and a change in kidney size is an important factor for evaluating renal condition in patients with kidney disease [1,2,3]. In chronic kidney disease, the kidney’s size decreases with disease progression due to a reduction in nephron mass, whereas in polycystic kidney disease, it increases with the functional decline due to the growth of cysts [4,5,6]. Therefore, reliable reference data based on accurate kidney size measurements are essential for evaluating the course of renal disease and anomalies in children [7,8,9]. The renal length shows a linear correlation with the kidney size and is usually used as a clinical indicator of kidney size changes due to the simplicity of the measurement. However, given that kidney length is a poor predictor of renal parenchymal volume and renal function, kidney volume is a better indicator of kidney size change [10,11,12].

There are several methods for measuring kidney volumes, such as computed tomography (CT), magnetic resonance imaging (MRI), and ultrasonography (US) [13]. Even though CT and MRI provide greater accuracy than US, they have limitations for clinical use in children due to sedation and exposure to radioactivity [14,15,16]. Therefore, US is the most appropriate method for children’s studies since it is relatively reliable and noninvasive [16,17]. The ellipsoidal method was a common kidney volume US measurement method in children, based on the ellipsoidal equation (kidney volume = depth × width × length × π/6) [17]. However, since this equation is based on geometric assumptions, the calculation does not reflect the actual kidney size. Previous studies have reported an underestimation of kidney volume when using this equation [15,18].

A new US-based kidney volume measurement method that reflects the actual kidney structure was necessary to overcome the problems of the ellipsoidal equation. Some previous studies have attempted the image segmentation process [19,20] using cross-sectional US images to calculate the kidney volume. A manual calibration process is essential for the segmentation of US images; however, it is time-consuming, labor-intensive, and highly prone to inter-observer variability. For these reasons, automatic segmentation methods for US kidney images using artificial intelligence have been proposed in previous studies [21,22,23,24,25]. Recently, hybrid learning, which combines deep learning and machine learning, has been applied as a learning method that can increase the accuracy of the automated segmentation process [26]. Thus, we combined the deep learning-based U-net model and the machine learning-based localizing region-based active-contour method for hybrid learning. To our knowledge, no previous study has proved that an automated segmentation process using US images could calculate the exact kidney volume in children by hybrid learning compared to the reference volume obtained by CT or MRI. We applied hybrid learning to automated segmentation and calculated the kidney volume with automatically segmented US images.

The purpose of this study was to develop an advanced automated method for accurate kidney volume measurement using US image segmentation and to establish an equation for the expected kidney volume (EKV) in healthy children. We have automated this method by applying artificial intelligence-based hybrid learning (deep and machine learning) and validated the accuracy of the automated segmentation. We have also proved the reliability of the kidney volume calculation using the proposed process by comparing it to the gold standard kidney volume measurement methods using CT or MRI.

## 2. Materials and Methods

### 2.1. Subject Analysis

This retrospective study was approved, and the requirement for informed consent was waived by the Gangnam Severance Hospital, Institutional Review Board (IRB No. 3-2020-0079). Patient records and information were anonymized and deidentified before analysis. This study was carried out in accordance with the Gangnam Severance Research Policies, the Bioethics and Safety Act, the International Conference on Harmonization (ICH) guidelines and the Declaration of Helsinki. US images of subjects under 19 years of age who visited the Gangnam Severance Hospital from July 2006 to February 2020 were reviewed. Among the subjects who underwent abdominal US to screen for abdominal disease, those who showed decreased renal function, abnormal urinalysis findings, renal parenchymal abnormalities, and anomalies in the kidney and urinary tract were excluded from the study.

Using US images, the volumes of 282 kidneys (141 left and 141 right kidneys) in 141 subjects were measured. The average age of the subjects was 6.3 years. The subjects were divided into the following groups based on age: 0–5 years (48.9%), 6–12 years (32.6%), and 13–18 years (18.4%). The ratio of boys to girls was 59:41. Of the 141 subjects, kidney US and CT were performed simultaneously in 29 subjects (Table 1).

### 2.2. Statistical Analysis

Statistical analyses were performed using SPSS (version 25; IBM, Armonk, NY, USA). Kidney volumes were compared based on sex and side using the paired t-test. Univariate regression analysis was applied to evaluate the relationships between kidney volume and the various parameters affecting it. Multivariate regression analysis was used to develop the EKV equation. The accuracy of each volume measurement method was evaluated based on ICCs.

### 2.3. Kidney Volume Measurement

#### 2.3.1. US Images and Ellipsoidal Method

**Figure 1:**
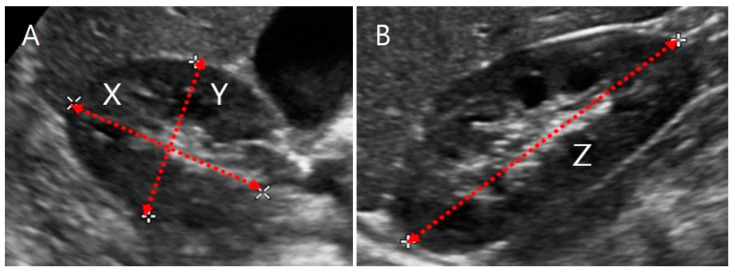
Ultrasound image of a kidney. (**A**) Transverse section. X is the maximum depth of the kidney parallel to the hilum, and Y is the maximum width perpendicular to X. (**B**) Coronal section. Z is the maximum bipolar length.

In the ellipsoidal method, the three orthogonal axes of the kidney were measured by US, and the kidney volume was calculated using the ellipsoidal equation [27]:

ELLIP_Vol = depth (X) (cm) × width (Y) (cm) × length (Z) (cm) × π/6,

where the length of the kidney (Z, maximum bipolar length) was measured in the coronal plane, while the depth (X, maximum length parallel to the hilum) and the width (Y, maximum length perpendicular to X) were measured in the transverse hilar region (Figure 1).

#### 2.3.2. US Images and Image Processing Program

MATLAB is multi-purpose engineering image processing software [28]. We designed the volume measurement process with this software to segment the kidney’s border based on the contrast gradient in the US images by the active contour method and to calculate the kidney volume [29].

The kidney boundaries were manually drawn by a pediatric nephrologist with 20 years of experience to obtain the ground truth of the kidney US image. The kidneys have a smooth appearance and clear boundaries with the surrounding blood vessels and organs; hence, it is straightforward to draw an accurate outline manually from two-dimensional (2D) US images. However, in some cases, when the kidney boundaries were ambiguous, they were determined in consideration of the anatomical appearance of the normal kidney. The starting point of the ureter from the hilum was the area where the inter-observer variability during manual editing may have increased. Therefore, we defined some calibration standards for this region. In the transverse section of the hilar region, the outline was manually drawn by connecting two contact points where the ureter emerges from the hilum. The coronal section was obtained by turning the transducer 90° at the transverse plane, and the kidney outline was manually drawn based on the contrast gradient and anatomical appearance of the normal kidney. The kidney boundaries were primarily determined by a pediatric nephrologist and then re-evaluated by a pediatric radiologist with 25 years of experience. The manually edited kidney structure was segmented by the active contour method. In the case of kidneys with an abnormal structure, inter-observer discrepancies may have occurred because it is challenging to identify the kidney boundaries accurately in areas with an unclear outline. However, we assume that the errors were minimized because only cases with a typical kidney structure, as confirmed by US, were included in this study.

**Figure 2:**
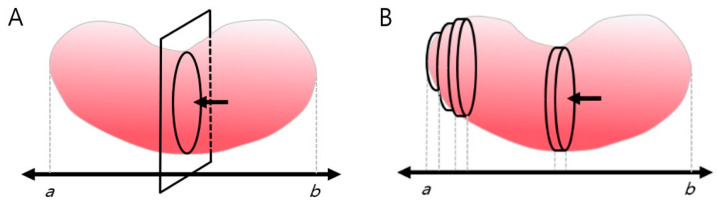
Measurement of kidney volume using an image processing program. (**A**) The central renal transverse section’s area captured by ultrasonography was segmented and calculated using MATLAB. (**B**) The transverse plane was automatically integrated along the coronary plane of the kidney and the length of the long axis (ab). One-way arrow(←) indicates mid cross-section of hilar region.

The number of pixels inside the kidney border was summed up to calculate the transverse section’s area automatically. Since the kidney’s hilum is anatomically likely to be located near its center [30], the hilar region was designated as the standard point for calculating the transverse section area. After calculating the area of the hilar region’s transverse section based on image segmentation, the area along the longitudinal axis (ab) of the coronal section was integrated to reconstruct the three-dimensional (3D) shape of the kidney, and its volume (IMGSEG_Vol) was calculated (Figure 2).

#### 2.3.3. CT Images and Volume Calculation

We determined the kidney volume (CT_Vol) by CT and a volume-calculating program (Aquarius iNtuition Viewer; TeraRecon, Durham, NC, USA). CT_Vol was defined as the standard reference volume. Aquarius iNtuition viewer is helpful software for volumetric analysis; previous studies have used it for several organs, such as the liver, heart, and kidney [31,32,33,34].

### 2.4. Automation of Volume Measurement by Hybrid Learning

The kidney’s cross-sectional area, according to the contrast gradient in a US image, can be extracted using active contour image segmentation by machine learning. Since the contrast gradient is often unclear along the kidney borders due to the characteristics of US images, the border should be determined through manual calibration. Thus, we applied hybrid learning to automate the image segmentation process, which combines machine learning and deep learning to maximize the function of the conventional machine and deep learning [26,35,36].

#### 2.4.1. Datasets

Both renal US and CT imaging were performed in 29 subjects. The renal segmentation dataset is consisted of the coronal and transverse sections, and the left and right kidneys were not distinct. The details are reported in Table 2.

The coronal plane data included 173 patients and 326 data points. The transverse plane data included 175 patients and 327 data points. The data were divided into train, validation, and test sets with an 8:1:1 ratio. The data from the 29 patients who underwent both US and CT imaging were placed into the validation and test sets.

#### 2.4.2. Data Augmentation Using Thin-Plate Spline Transformation

We had relatively little data on the transverse and coronal kidney US images to apply to the deep learning model; therefore, we applied thin-plate spline (TPS) transformation to increase the data [37]. The TPS model is mainly used for image transformation and shape matching. It is a spline interpolation method that allows for adjusting the smoothing level and calculating the coordinate transformation coefficient for any point. This is performed using a moving image M and a fixed image F. For example, given an image of the kidney US in the coronal plane, image M is registered in image F to generate a transformed image.

The TPS model used for a 2D coordinate transformation can be expressed as follows:

(1) $$f_{x^{\prime }}\;\left(x,y\right)=a_{1}+a_{x}x+a_{y}y+\overset{p}{\underset{i=1}{\sum }}\omega_{xi}U\left(\|\left(x_{i},y_{i}\right)-\left(x,y\right)\|\right)\;\;$$

(2) $$f_{y^{\prime }}\;\left(x,y\right)=b_{1}+b_{x}x+b_{y}y+\overset{p}{\underset{i=1}{\sum }}\omega_{yi}U\left(\|\left(x_{i},y_{i}\right)-\left(x,y\right)\|\right)\;\;$$

$\left(x_{i}^{’},\;y_{i}^{’}\right)$ represents the target function value in the $\left(x_{i},\;y_{i}\right)$ planes where i=1, 2,…, p. It is assumed that the positions $\left(x_{i},\;y_{i}\right)$ are all different and are not collinear. $a_{1},\;a_{x},\;a_{y},\;b_{1},\;b_{x},\;b_{y},\;\omega_{xi},\;\omega_{yi}$ represents the transformation parameter and $U\left(r\right)=r^{2}\log r$. For f(x, y) to have a squared integral second derivative, we need the following:

(3) $$\overset{p}{\underset{i=1}{\sum }}\omega_{i}=0\;and\;\overset{p}{\underset{i=1}{\sum }}\omega_{i}x_{i}=\overset{p}{\underset{i=1}{\sum }}\omega_{i}y_{i}=0$$

First, the kidney boundary of the ground truth image of the moving image M and the fixed image F is modeled and approximated as an ellipse. It is then identified as the feature points of the four vertices of the ellipse of the moving image M and the fixed image F. Then, the TPS operation O is obtained by registering the feature points of the moving image M to the feature points of the fixed image F. The landmarks of the moving image M and the fixed image F are represented by $Q_{M}={\left[x_{i},\;y_{i}\right]}^{T}\in R^{2\times 4}$ and $Q_{F}={\left[x_{i}^{’},\;y_{i}^{’}\right]}^{T}\in R^{2\times 4}$, respectively. TPS operation O is defined as follows:

(4) $$O=\left[\begin{array}{c} \omega^{4\times 2}\\ a^{3\times 2} \end{array}\right]={\left[\begin{array}{cc} K & P^{T}\\ P & L^{3\times 3} \end{array}\right]}^{-1}\left[\begin{array}{c} Q_{M}^{T}\\ L^{3\times 2} \end{array}\right]$$

**Figure 3:**
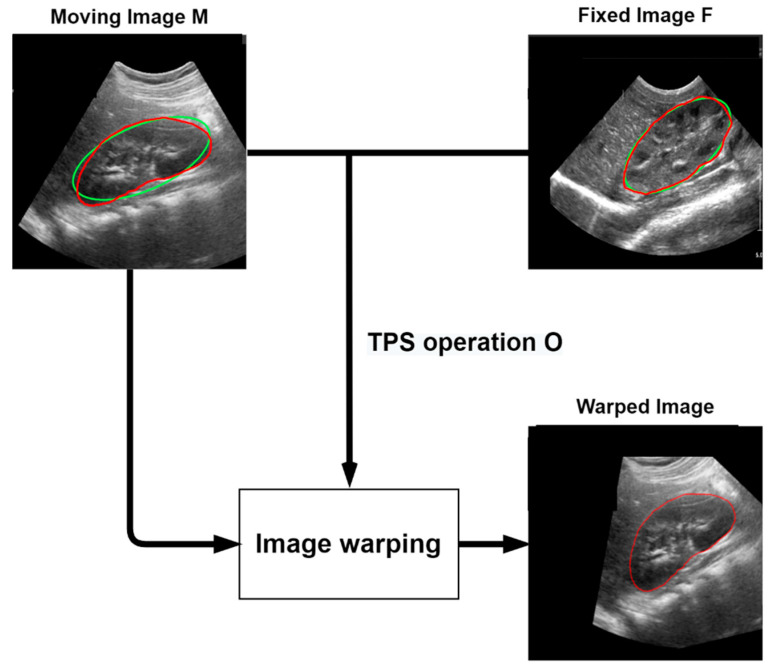
The TPS transformation process. The moving image M is transformed using the TPS parameters obtained from the relationship between the moving image M and the fixed image F. Green line: Ellipse Approximate to Ground Truth, Red line: Ground Truth.

$K_{ij}=U(\|\left(x_{i},\;y_{i}\right)-\left(x_{j},y_{j})\right\|)$ and $P={\left[\begin{array}{cc} I^{4\times 1} & Q_{M}^{T} \end{array}\right]}^{T}\in R^{3\times 4}$ is the homogeneous coordinate of $Q_{M}$, and a is the column vector with elements $a_{1},\;a_{x},\;a_{y},\;b_{1},\;b_{x},\;b_{y}$. $L^{3\times 3}$ is a 3×3 matrix of zeros, and $L^{3\times 2}$ is a 3×2 vector of zeros. A warped image was created using the calculated TPS parameter. Given n training images, we can obtain k(k−1)/ratio)+k augmented training images with boundaries, where k is the number of raw train datasets. To prevent the data from increasing too much, the ratio was set to 16 in the coronal and transverse trainset images. Figure 3 shows the above TPS transformation process.

#### 2.4.3. Deep Learning Network and Loss Function

U-net is an artificial neural network that was proposed for deep learning to deal with medical image segmentation [38]. This architecture is called U-net because it has a U-shaped structure. For accurate localization, it is composed of a network to obtain the overall context information of an image and a symmetrical network. There is an encoding part for extracting the features of the image and a decoding part for detailed localization. The up-convolution feature map for each decoding step is concatenated with the encoding step’s cropped feature map to segment multi-scale objects effectively. The model we used was constructed by modifying the bottom part of the conventional U-Net. It was fixed to 512 channels at the bottom and reduced to a quarter of the connected channels.

The loss function calculates the difference between the resulting image obtained through the deep learning model and the ground truth image and then updates the weights to reduce the loss. We combined the following three loss functions (BCEwithLogitsLoss, dice loss, and focal loss) to get a more precise stretch segmentation result [39].

BCEwithLogitsLoss is a combination of BCE loss and sigmoid layer and can be expressed as follows:

(5) $$\begin{array}{c} \ell_{bce}\left(p,\;y\right)=L={\left\{l_{1},\ldots ,\;l_{N}\right\}}^{T},\\ l_{n}=-\omega_{n}\left[y_{n}\cdot \log\sigma \left(p_{n}\right)+\left(1-y_{n}\right)\cdot \log\left(1-\sigma \left(p_{n}\right)\right)\right] \end{array}$$

y is the ground truth, p has a value between 0 and 1, and the probability of y=1 is estimated from the model. N is the batch size, n is the number of the sample in the batch, and σ is the sigmoid function.

The dice score coefficient is a widely used overlapping measure to evaluate the segmentation performance when the ground truth is available. The dice loss can be expressed as the following:

(6) $$\begin{array}{l} Dice\;coefficient=\frac{2\left|A\;\cap \;B\right|}{\left|A\right|+\left|B\right|},\\ \ell_{dice}=1-Dice\;coefficient \end{array}$$

|A∩B| represents the common element between sets *A* and *B*, |A| represents the number of elements in set *A* (also for set *B*). |A∩B| is computed as a pixel-wise multiplication between the prediction result and the ground truth.

Focal loss is introduced as an extension of cross-entropy loss designed for focused learning of hard-to-classify parts by lowering the weight on easily classified parts. It is defined as follows:

(7) $$CE\left(p,y\right)=\{\begin{array}{c} \begin{array}{cc} -\log\left(p\right) & if\;y=1 \end{array}\\ \begin{array}{cc} -\log\left(1-p\right) & otherwise \end{array} \end{array}$$

Here, regardless of y, $p_{t}>0.5$ significantly reduces the loss value due to high model confidence, but the problem is easily classified and tends to exceed 0.5 and reduces the loss value too much. This can overwhelm the impact of difficulty to classify on loss. Therefore, a weighting factor α was proposed. When y=−1, the weight of (1−α) is given to loss, and when y=1, the weight of α is given. Cross-entropy with a weighting factor added is expressed as follows:

(8) $$\begin{array}{c} CE\left(p,y\right)=-\alpha_{t}\log\left(p_{t}\right)\\ \mathrm{If}\;\alpha =0.7,\;\mathrm{it}\;\mathrm{can}\;\mathrm{be}\;\mathrm{expressed}\;\mathrm{as}\;\mathrm{follows}:\\ CE\left(p,y\right)=\{\begin{array}{c} \begin{array}{cc} -0.7\log\left(p\right) & if\;y=1 \end{array}\\ \begin{array}{cc} -0.3\log\left(1-p\right) & otherwise \end{array} \end{array} \end{array}$$

The weighting factor α was used to adjust the effect of positive and negative samples on losses, but the degree of reflection of losses on easy or hard samples was not adjusted. This is resolved by the following scaling factor ${\left(1-p_{t}\right)}^{\gamma }$, which is called focal loss [40].

(9) $$\begin{array}{l} \ell_{focal}\left(p_{t}\right)=-{\left(1-p_{t}\right)}^{\gamma }\log\left(p_{t}\right),\\ \ell_{focal}\left(p,y\right)=\{\begin{array}{c} \begin{array}{cc} -{\left(1-p\right)}^{\gamma }\log\left(p\right) & if\;y=1 \end{array}\\ \begin{array}{cc} -{\left(1-\left(1-p\right)\right)}^{\gamma }\log\left(1-p\right) & otherwise \end{array} \end{array} \end{array}$$

where γ is the focus parameter that controls weight reduction in easily classified examples. When γ=0, the focal loss is equal to the cross-entropy loss. As the value of γ increases, the greater the value of p, and the smaller the loss function.

We were able to acquire a more precise segmentation result by combining the three loss functions mentioned above.

(10) $$\ell =a\cdot \ell_{bce}+b\cdot \ell_{dice}+c\cdot \ell_{focal}$$

Pixel-wise classification with BCE loss, shape adjustment with dice loss, and data imbalance problem with focal loss has been improved.

**Figure 4:**
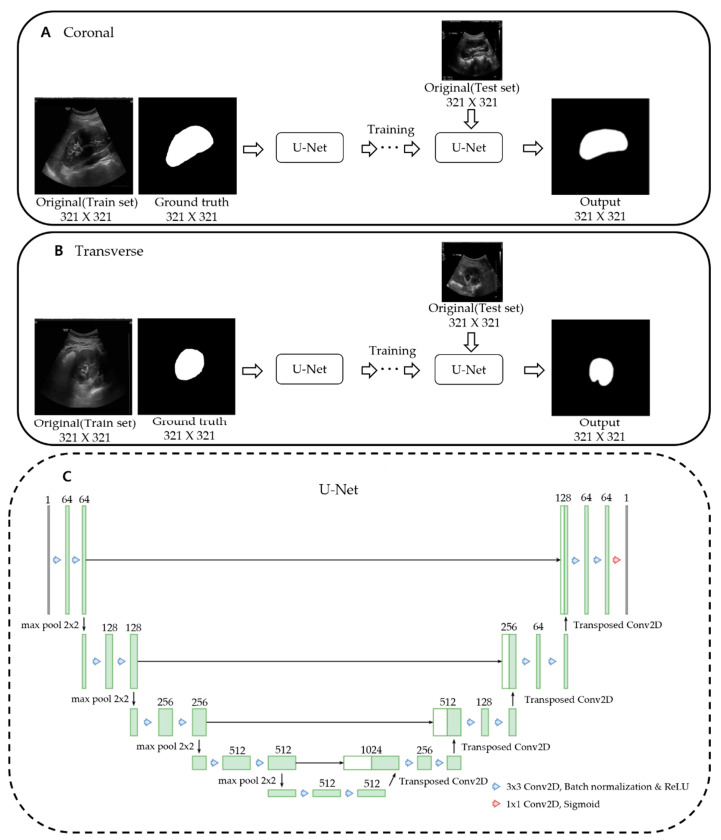
Training of a U-Net model. U-Net models for coronal and transverse images were trained separately. (**A**) Input data for the U-Net model are original and ground truth coronal images of the training dataset. After training, output images are obtained from the original coronal images of the test dataset via trained U-Net. (**B**) The same process as above is repeated in transverse images. (**C**) Architecture of U-Net training model. We modified the bottom part of the conventional U-Net. It was fixed to 512 channels at the bottom and reduced to a quarter of the connected channels.

Figure 4 shows the training of a U-Net model with an augmented dataset using TPS transformation. We trained the U-Net models for coronal and transverse images separately.

**Figure 5:**
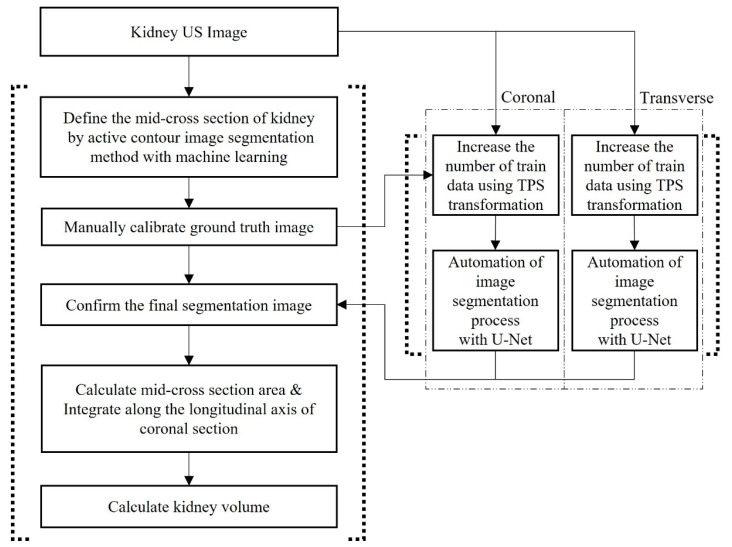
Flowchart of the automated volume measurement process with hybrid learning. US: Ultrasonography.

We repeatedly input the original US image and manually calibrated ground truth images into U-net. U-net automatically segmented the images to produce the final segmentation images without manual calibration. After that, the image segmented through U-net was set as the initial mask of the localizing region-based active contour model, resulting in smoother results [29]. Thus, we combined the deep learning-based U-net model and the machine learning-based localizing region-based active contour method for hybrid learning so that the image processing program could determine kidney borders automatically without manual calibration (Figure 5).

We trained the model on each dataset of the coronal and transverse sections using the loss function described above and weighted BCE, dice, and focal loss by 0.8, 1.0, and 1.0, respectively. The image has been resized to a size of 321 × 321. An Adam optimizer was used as the optimizer. The learning rate was set to 5e-4, and an early-stopping technique was applied to prevent overfitting. In addition, the focus parameter γ of focal loss was set to 0.5.

## 3. Results

### 3.1. Comparison of Kidney Volumes Based on Sex, Age, and Position of the Kidney

Paired *t*-tests were performed to compare the average kidney volume measured by image segmentation (IMGSEG_Vol) between left and right kidneys in each age group and sex. The age groups 0–5, 6–12, and 13–18 years showed no significant volume differences between the right and left kidneys (*p* > 0.05). In addition, there were no significant sex differences among the right or left kidney volumes (*p* > 0.05, Table 3).

### 3.2. Correlation between Age and Kidney Volume Measured by Different Methods

**Figure 6:**
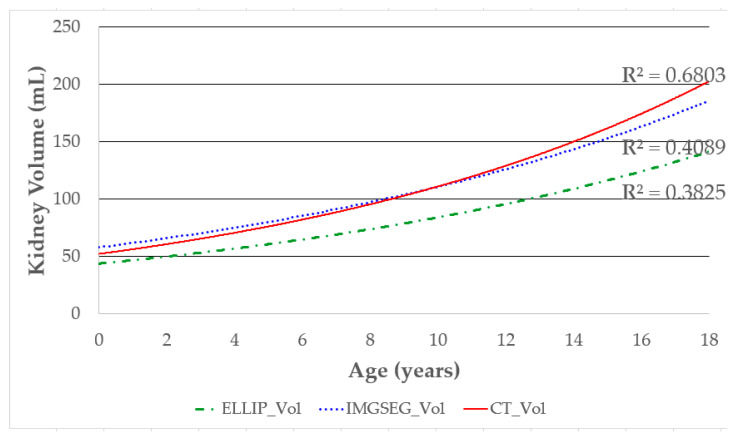
Correlation between age and kidney volume according to volume measurement methods. The R^2^ values are shown to compare the correlation between age and kidney volume obtained by each volume measurement method. Higher R^2^ values indicate a stronger correlation.

The correlation between age and kidney volume was compared based on the measurement method. The kidney volume by CT (CT_Vol) showed the highest correlation with age (R^2^ = 0.6803), followed by IMGSEG_Vol (R^2^ = 0.4089) and volume, measured with the ellipsoidal method (ELLIP_Vol, R^2^ = 0.3825; Figure 6).

### 3.3. Degree of Agreement with the Reference Kidney Volume

The intraclass correlation coefficients (ICCs) indicate the degree of agreement between the reference kidney volume (CT_Vol) and those calculated by the different methods. The ICCs for IMGSEG_Vol and ELLIP_Vol were 0.909 (95% CI, 0.847–0.946) and 0.805 (95% CI, 0.327–0.919), respectively, which were significantly different (*p* < 0.05) (Table 4).

### 3.4. Factors Affecting Changes in Kidney Volume

The correlation between IMGSEG_Vol with various factors affecting kidney volume changes was first verified by univariate regression analysis (Table 5).

The weight (R^2^ = 0.809), body surface area (BSA; R^2^ = 0.792), height (R^2^ = 0.724), age (R^2^ = 0.690), and body mass index (BMI; R^2^ = 0.386) were all significantly correlated with IMGSEG_Vol (*p* < 0.001). Next, the weight and height, which showed the most significant correlations with IMGSEG_Vol, were used in multivariate regression analysis (Table 6).

We also formulated the following equation to estimate the EKV:

EKV = [2.22 × weight (kg) + 0.252 × height (cm) + 5.138](11)

**Figure 7:**
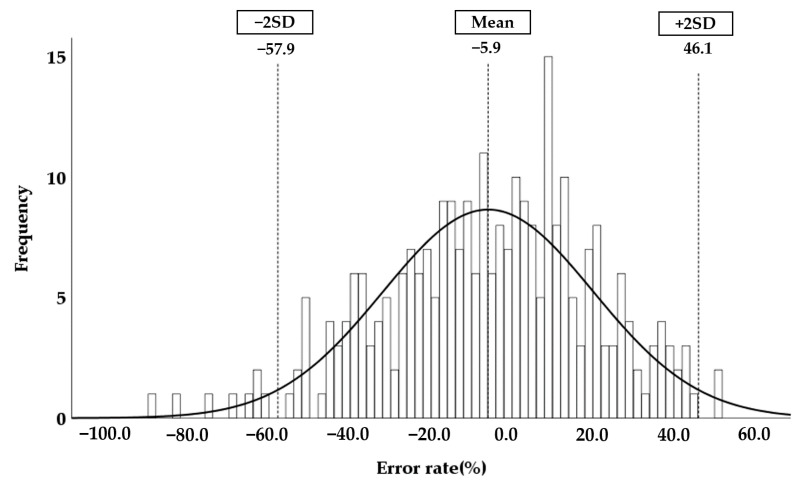
Normal distribution of the error rate. The error rate was calculated according to Equation (12). The mean error rate and ± double standard deviation (2SD) are plotted on the normal distribution graph.

The error between IMGSEG_Vol and the calculated EKV was determined. The mean error rate was −5.9%, and the range of the mean error rate ± 2 SD was −57.9 to 46.1% (Figure 7).

(12) $$\mathrm{Error}\;\mathrm{rate}\;(\%)=\frac{\left(\;\mathrm{measured}\;\mathrm{IMGSEG}\_\mathrm{Vol}\;–\;\mathrm{calculated}\;\mathrm{EKV}\;\right)}{\mathrm{measured}\;\mathrm{IMGSEG}\_\mathrm{Vol}}\times 100$$

### 3.5. Accuracy of the Automatically Measured Kidney Volume Using Hybrid Learning

The results of the child kidney segmentation were evaluated quantitatively, as shown in Table 7. The segmentation results were compared by recall, precision, and F1 score.

Recall is the rate at which a model correctly predicts the true answer. It is also commonly used to measure sensitivity and hit rate. The formula is

(13) $$\mathrm{Recall}=\frac{\mathrm{TP}}{\mathrm{TP}+\mathrm{FN}}=\frac{I_{\mathrm{pred}}\cap I_{\mathrm{GT}}}{\left|I_{\mathrm{GT}}\right|}$$

Precision is the proportion of what the model classifies as true is actually true. This is also called the positive predictive value (PPV). This is expressed as follows:

(14) $$\mathrm{Precision}=\frac{\mathrm{TP}}{\mathrm{TP}+\mathrm{FP}}=\frac{I_{\mathrm{pred}}\cap I_{\mathrm{GT}}}{\left|I_{\mathrm{pred}}\right|}$$

The F1 score is the harmonic mean of precision and recall, and is expressed as follows:

(15) $$F1_{\mathrm{score}}=\frac{1}{\frac{1}{\mathrm{Precision}}+\frac{1}{\mathrm{Recall}}}=2\times \frac{\mathrm{Precision}\times \mathrm{Recall}}{\mathrm{Precision}+\mathrm{Recall}}\;$$

The F1 score can accurately evaluate the performance of the model when the data labels are unbalanced, and the performance can be expressed as a single number. This is also known as the dice score.

The kidney segmentation study was conducted on each dataset on the coronal and transverse planes. In the coronal plane, the three evaluation indicators exceeded 90%, and the segmentation results were excellent. In the transversal plane, the results approached 90%. The trained hybrid learning model performed better on the coronal images than the transverse images in the validation process using recall, precision, and the F1 score(Table 7).

**Figure 8:**
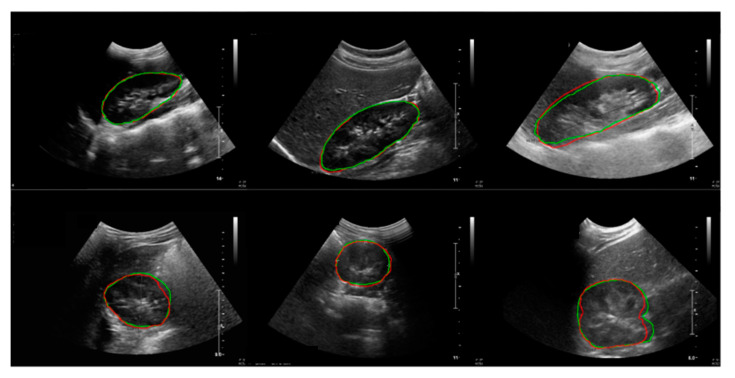
Results of the kidney segmentation. Red line: model prediction; green line: ground truth. First row: coronal plane; second row: transverse plane.

The results of the child kidney segmentation were evaluated qualitatively, as shown in Figure 8. The segmented outlines of the kidney images of the ground truth and deep learning results show good accordance on the coronal and transverse planes.

**Figure 9:**
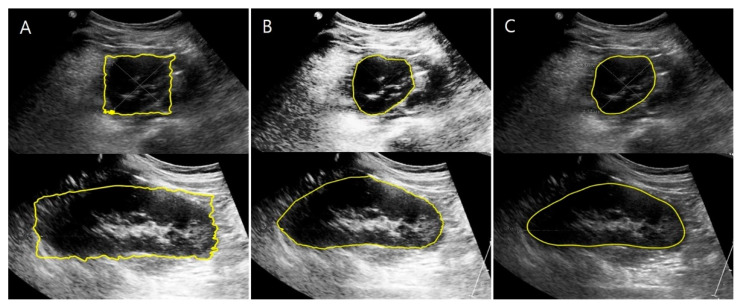
Comparison of kidney boundaries based on the stage of segmentation. (**A**) Kidney boundaries extracted without manual calibration. (**B**) Kidney boundaries obtained using manual calibration. (**C**) Kidney boundaries extracted automatically using hybrid learning.

Before the manual calibration, only the square-shaped regions of interest were determined, and the results show inaccurate kidney boundaries (Figure 9A). Therefore, manual calibration with accurate kidney boundaries was performed to obtain the ground truth images (Figure 9B). The kidney boundaries were automatically extracted with the proposed hybrid learning method using segmented kidney US images from U-Net as an initial mask for the active contour processing (Figure 9C).

The ICC for HYBRID_Vol was 0.925 (95% CI, 0.872–0.956), which was significantly different from that for ELLIP_Vol (*p* < 0.01). The ICC for IMGSEG_Vol was 0.909 (95% CI, 0.847–0.946), which was also significantly different from that for ELLIP_Vol (*p* < 0.05). There were no significant differences in the ICCs for HYBRID_Vol and IMGSEG_Vol (*p* = 0.59) (Table 8).

## 4. Discussion

Childhood is a crucial period of growth for many organs. Age and kidney size are closely related to renal function. Therefore, an age-matched normal reference value of kidney volume can help in the diagnosis and prognosis of kidney diseases [8,9,41]. In children, an accurate estimation of the EKV is necessary, and an age-matched normal kidney growth curve is the ideal method to evaluate kidney size. In children, US is the preferred method for measuring kidney volume because it is safer and simpler than CT and MRI.

Although 3D US can be advantageous in defining the outer appearance of the kidney, 2D US is still widely used for kidney volume measurement. 3D US has the disadvantages of high costs, complicated computational processes, and limitations in evaluating the renal parenchyma due to the low resolution [42,43]. For these reasons, evaluation of the kidney parenchyma should start with 2D US before measuring the kidney volume in children by 3D US.

3D US has complicated computational processes because it reconstructs the outer kidney appearance with multiple parallel cross-sectional images, similar to the CT and MRI methods. On the other hand, the proposed method using MATLAB on 2D US is relatively inexpensive, and the computational burden of programming is lower than that of 3D US since the MATLAB-based volume measurement methods in this study use only a single mid-transverse and one coronal image. In addition, when artificial intelligence is applied to 3D US, training a deep network on a large dataset may be more computationally expensive for real clinical applications than 2D US [20,44]. Furthermore, 2D US is preferred for clinical research because it is widely used as the conventional screening imaging study method for kidney disease and is advantageous for data accumulation.

Kidney volume calculated by the ellipsoidal equation is frequently used to compare them with measurements done by 2D renal US. However, the ellipsoidal method usually underestimates kidney volume by 15–25% compared to CT or MRI measurements [15,18,27]. Therefore, we have proposed an advanced method for kidney volume measurement using 2D renal US and MATLAB to overcome the limitations of the current evaluation methods. In previous studies, the inaccuracy of the ellipsoidal method was corrected by multiplying a uniform constant to resolve the problem of underestimation [27]. However, such a uniform correction could not overcome the limitation of the ellipsoidal method because of the wide range of underestimation errors associated with it [15].

Furthermore, the ellipsoidal equation requires manual measurements of the cross-sectional area’s width and depth for calculating the kidney volume, which is prone to errors. In contrast, our proposed method does not require such manual measurements because the cross-sectional area and kidney volume are calculated automatically from US images, which allows for more accurate measurements. Our method is partly based on the stepped section method reported by Rasmussen et al. [19]. However, the stepped section method requires serial parallel transverse kidney US images to calculate the kidney volume, making it time consuming and impractical, especially in children. In contrast, our method uses a single mid-transverse image and demonstrates a significantly higher degree of agreement (ICCs) of IMGSEG_Vol with the reference volume (CT_Vol) compared to the ellipsoidal method (*p* < 0.05) (Table 4).

To develop the EKV equation for children, the variables that affect kidney volume (IMGSEG_Vol) need to be evaluated. It is well-known that weight, height, age, and BSA are highly correlated with renal volume, of which weight and height are the most significant [7,8,17,45]. Our results also show that weight and height were the most reliable predictors of EKV, and therefore, we used these parameters in the EKV equation. While some previous studies have reported differences in kidney size based on sex and side [7,8,45], we and others found no significant differences based on these factors [9,46]. Therefore, we did not consider sex or the side the kidney is on when formulating the EKV equation.

To ensure accurate kidney volume (IMGSEG_Vol) measurements, they should be compared with the reference volume calculated using the EKV. Previous studies used the mean ± SD or the mean ± 2 SD to assess the reference range for the normal kidney size [1,8]. Clinical renal hypoplasia is diagnosed when the kidney volume is less than the mean —2SD [4]. We defined the mean error rate ± 2 SD as the cut-off range to determine the measured kidney volume’s accuracy and found that the cut-off ranged from −57.0% to +45.7%. A measured volume outside of the cut-off range indicates that it is more or less than the normal average kidney volume. We expect this cut-off range to be a predictor of kidney disease progression and an indicator for further clinical investigation.

The assessment of kidney size using the EKV or the expected kidney length is based on the linear proportional relationship between kidney volume and variables such as weight and height [1,17]. Ultimately, the gold standard method for kidney size assessment in children involves comparison with a real reference volume (normal kidney) in an age-matched manner, as in a fitted centile growth chart for kidney volume, which has been reported only for fetal stages so far [47,48]. To develop such a fitted centile growth chart for children, data on kidney volumes for different age groups should be collected from various countries or ethnic regions to reflect all differences. For these reasons, volume measurement should be accurate, simple, and with minimal inter-observer error.

Kidney volume measurement using an image processing program requires the manual calibration of image segmentation to define the kidney’s boundary accurately in US images [49]. Manual calibration is highly dependent on human experience, which is time consuming and prone to inter-observer variability. To overcome this problem, we applied hybrid learning to the image processing program. Hybrid learning is an artificial intelligence-based learning technology that combines machine learning and deep learning. Combined with the image processing program, hybrid learning enabled the automatic segmentation and calculation of kidney volume (HYBRID_Vol) directly from the US image without manual calibration (Figure 9).

Deep learning requires several datasets. Because our dataset was relatively small for training deep learning models, the training results are limited. However, to address this problem, we improved the accuracy by generating more meaningful data through thin-plate spline (TPS) transformation rather than the classic method of using conventional image processing techniques to train the deep learning models [37]. U-net, a deep learning model optimized for medical image segmentation, was used [38]. The results of deep learning were applied to the active contour model to create smoother results. We also adopted the BCE, dice, and focal loss to assess the loss of function, reflecting information on the shape of the kidney and focusing on data that are difficult to learn through focal loss.

We independently trained each U-Net model on the coronal and transverse section datasets. We thought that separate training of each homogenous model could lower the complexity of the dataset by making the distribution of the dataset constant. Thus, we assumed that each model could learn efficiently to obtain the best results even if the number of datasets is small. There was a previous study in which a deep learning model was trained separately for each section of the hippocampus in the brain [50]. The segmentation results among the sagittal, coronal, and axial sections of the hippocampus were different and it seemed that the more complex structure showed worse performance.

The number of data was sparse in this study because obtaining the proper image data, especially from children, is difficult in a retrospective study. Therefore, we trained the U-Net model separately on the coronal and transverse sections to maximize the efficiency of the training. The outline of the transverse section is more complex than that of the coronal because of the region where the ureter emerges from the hilum. Our data show that the coronal section performed better than the transverse section because there were more conditions to be considered during the training for the segmentation of the transverse section. In consideration of the distinct differences of features between the coronal and transverse sections and the small number of data, we thought that the separate training of each section of the kidney was adequate for our study.

We found significant differences between the ICCs for HYBRID_Vol and ELLIP_Vol. The ICC for HYBRID_Vol was higher than that for IMGSEG_Vol (Table 8). Therefore, our findings demonstrate that this new automated method is more accurate than the ellipsoidal method, and that manual calibration can be successfully replaced by an automated process after hybrid learning. This is the first study on children to use image pre-processing and hybrid learning to determine kidney volume changes.

There are some limitations to our study. First, in retrospective studies, including ours, it is generally challenging to collect consistent US kidney images. The consistency of the cross-sectional images is an important factor for research with MATLAB and hybrid learning. In contrast, our radiology department already had an exact US protocol for deciding the transverse and coronal sections of the kidney that met our criteria; thus, we could minimize the difficulty in collecting consistent US images. Second, we had to analyze a limited number of images to avoid training bias because, in hybrid learning, imaging data for ICC evaluation should not be repeatedly used. More imaging data for hybrid learning would have improved the accuracy of HYBRID_Vol. Third, even if the model was further optimized using the TPS transformation, there was insufficient evaluation data to examine the potential bias, statistical uncertainty, and generalizability of the proposed model. Therefore, we aim to collect more data in the future for a more objective evaluation. Lastly, there was not enough kidney volume data for each age group to develop a fitted centile curve of kidney growth based on pediatric age. Therefore, instead of a kidney growth curve, we developed an equation to calculate the EKV and decided on a cut-off range (mean error rate ± 2 SD). Our future research will focus on developing fitted centile growth curves based on pediatric age, with large amounts of prospectively collected HYBRID_Vol data using our new automated method.

## 5. Conclusions

We propose a new advanced automated method for kidney volume measurement using US image segmentation and established an equation for the expected kidney volume (EKV) in healthy children. We successfully automated this method by applying artificial intelligence-based hybrid learning. In addition, we proved the accuracy of automated segmentation and the reliability of kidney volume calculation using the proposed process by comparing it with the gold standard kidney volume measurement method. We propose this method to help develop an age-specific kidney growth curve by accumulating and analyzing large 2D US image datasets. It could also help to evaluate various kidney diseases by measuring the volume of pathological kidney structures, such as hydronephrosis, polycystic kidney disease, and renal tumors.

## Figures and Tables

**Table 1:** Classification and distribution of subjects.

	Group	No. of Subjects	(%)
Sex	Boys	83	(58.9)
	Girls	58	(41.1)
	Total	141	(100.0)
Age group (years)	0–5	69	(48.9)
	6–12	46	(32.6)
	13–18	26	(18.4)
	Total	141	(100.0)
Imaging study	US only	112	(79.4)
	CT + US	29	(20.6)
	Total	141	(100.0)

CT: computed tomography; US: ultrasonography.

**Table 2:** Datasets (coronal and transverse planes).

	Coronal Plane		Transverse Plane	
	No. of Data Points	No. of Subjects	No. of Data Points	No. of Subjects
Train	255	137	256	139
Validation	36	18	36	18
Test	35	18	35	18
Total	326	173	327	175

**Table 3:** Comparison of kidney volumes based on the position of the kidney, age, and sex.

**Age Group (years)**	**IMGSEG_Vol**		***p*-Value**
	**Right Kidney**	**Left Kidney**	
	**Mean (Error Measure)**	**Mean (Error Measure)**	
0–5	44.8 ± 23.0 ^†^	45.6 ± 22.7 ^†^	0.526 ^†^
6–12	109.0 ± 36.9 ^‡^	108.7 ± 36.6 ^‡^	0.932 ^‡^
13–18	170.3 ± 49.6 ^††^	161.3 ± 49.1 ^††^	0.636 ^††^
**Position of Kidney**	**IMGSEG_Vol**		***p*-Value**
	**Boys**	**Girls**	
	**Mean (Error Measure)**	**Mean (Error Measure)**	
Right	92.1 ± 63.1 ^∥^	84.3 ± 52.2 ^∥^	0.135 ^∥^
Left	90.8 ± 57.6 ^¶^	82.8 ±53.8 ^¶^	0.402 ^¶^

IMGSEG_Vol: Kidney volume determined by image segmentation. ^†^ *p* = 0.526: *p*-Value between right and left mean IMGSEG_Vol in age group 0-5 years. ^‡^ *p* = 0.932: *p*-Value between right and left mean IMGSEG_Vol in age group 6–12 years. ^††^ *p* = 0.636: *p*-Value between right and left mean IMGSEG_Vol in age group 13–18 years. ^∥^ *p* = 0.135: *p*-Value between boys and girls mean IMGSEG_Vol in right kidney. ^¶^ *p* = 0.402: *p*-Value between right and left mean IMGSEG_Vol in left kidney.

**Table 4:** Degrees of agreement among IMGSEG_Vol, ELLIP_Vol, and reference value (CT_Vol).

Kidney Volume	ICC *	95% CI **	*p*-Value
IMGSEG_Vol	0.909	0.847–0.946	*p* < 0.05
ELLIP_Vol	0.805	0.327–0.919	

IMGSEG_Vol: kidney volume measured by image segmentation; ELLIP_Vol: kidney volume measured by the ellipsoidal method; CT_Vol: kidney volume measured by computed tomography. * ICC: intraclass correlation coefficient; degree of agreement is higher as the ICC approaches 1.0. ** CI: confidence interval.

**Table 5:** Univariate regression analysis of factors affecting changes in kidney volume.

Independent Variables	Intercept	B *	Standard Error	Standardized Coefficient (β)	*p*-Value	$R^{2}$
Weight	20.599	2.467	0.079	0.899	*p* < 0.001	0.809
BSA	−2.878	106.281	3.277	0.890	*p* < 0.001	0.792
Height	−46.459	1.220	0.045	0.851	*p* < 0.001	0.724
Age	34.881	8.468	0.339	0.831	*p* < 0.001	0.690
BMI	−76.77	9.751	0.740	0.621	*p* < 0.001	0.386

* B: weight factor; BMI: body mass index; BSA: body surface area; IMGSEG_Vol: kidney volume measured by image segmentation.

**Table 6:** Multivariate regression analysis of variables affecting IMGSEG_Vol.

Independent Variables	Intercept	B *	Standard Error (SE)	Standardized Coefficient (β)	*p*-Value	VIF **	$R^{2}$
Weight Height	5.138	2.220 0.252	0.198 0.094	0.737 0.175	*p* < 0.001 *p* < 0.05	6.273	0.810

IMGSEG_Vol: kidney volume measured by image segmentation. * B: weight factor. ** VIF: variance inflation factor; multicollinearity, less than 10 means less interference between variables.

**Table 7:** Results of the kidney segmentation. This table shows the evaluation results for the test set of the model trained with each dataset of the coronal and transverse sections.

	Coronal Plane			Transverse Plane		
	Recall	Precision	F1 Score	Recall	Precision	F1 Score
Validation	0.9211	0.9716	0.9441	0.8617	0.9382	0.8808
Test	0.9310	0.9801	0.9538	0.8858	0.9224	0.8940

**Table 8:** Degree of agreement between HYBRID_Vol, IMGSEG_Vol, ELLIP_Vol, and reference value.

Method	ICC *	95%CI **	*p*-Value
HYBRID_Vol	0.925 ^‡^^,^^¶^	0.872–0.956	*p* = 0.59 ^‡^
IMGSEG_Vol	0.909 ^‡^^,^^∮^	0.847–0.946	*p* < 0.01 ^¶^
ELLIP_Vol	0.805 ^¶^^,^^∮^	0.327–0.919	*p* < 0.05 ^∮^

HYBRID_Vol: kidney volume measured by hybrid learning; IMGSEG_Vol: kidney volume measured by image segmentation; ELLIP_Vol: kidney volume measured by the ellipsoidal method; CT_Vol: kidney volume measured by computed tomography. * ICC: intraclass correlation coefficient; the degree of agreement is higher as the ICC approaches 1.0. ** CI: confidence interval. ^‡^ *p* = 0.59: *p*-Value between HYBRID_Vol ICC and IMGSEG_Vol ICC. ^¶^ *p* < 0.01: *p*-Value between HYBRID_Vol ICC and ELLIP_Vol ICC. ^∮^ *p* < 0.05: *p*-Value between IMGSEG_Vol ICC and ELLIP_Vol ICC.

## Data Availability

The datasets analyzed during the current study are available from the corresponding author upon reasonable request.
